# Identification, Molecular Characteristics, and Evolution of *GRF* Gene Family in Foxtail Millet (*Setaria italica* L.)

**DOI:** 10.3389/fgene.2021.727674

**Published:** 2022-02-03

**Authors:** Huilong Chen, Weina Ge

**Affiliations:** School of Life Science, North China University of Science and Technology, Tangsha, China

**Keywords:** growth-regulating factors, foxtail millet, structure, expression, loss

## Abstract

Growth-regulating factor (GRF) is a multigene family that plays a vital role in the growth and development of plants. In the past, the GRF family of many plants has been studied. However, there is not a report about identification and evolution of GRF in foxtail millet (*Setaria italia*). Here, we identified 10 *GRF* genes in foxtail millet. Seven (70.00%) were regulated by Sit-miR396, and there were 19 optimal codons in *GRF*s of foxtail millet. Additionally, we found that WGD or segmental duplication have affected *GRFs* in foxtail millet between 15.07 and 45.97 million years ago. Regarding the *GRF* gene family of land plants, we identified a total of 157 *GRF* genes in 15 representative land plants. We found that *GRF* gene family originated from Group E, and the *GRF* gene family in monocots was gradually shrinking. Also, more loss resulted from the small number of *GRF* genes in lower plants. Exploring the evolution of *GRF* and functional analysis in the foxtail millet help us to understand *GRF* better and make a further study about the mechanism of *GRF*. These results provide a basis for the genetic improvement of foxtail millet and indicate an improvement of the yield.

## Introduction

Growth-regulating factor (GRF) is a plant-specific transcription factor that plays an important role in plant growth and development. The first member of the identified GRF family is OsGRF1, which plays a regulatory role in gibberellin (GA)-induced stem elongation (van der Knaap et al., 2000). GRF transcription factor has two conserved domains in its N-terminal region: QLQ (Gln, Leu, and Gln) and WRC (Trp, Arg, and Cys) (Rodriguez et al., 2016). The QLQ domain interacts with GRF interacting factor (GIF), and the resulting complex acts as a transcriptional co-activator (Wang et al., 2014). The WRC domain consists of a functional nuclear localization signal and a DNA binding motif (zinc finger structure), which is mainly involved in DNA binding. The C-terminal of some GRF proteins also consists of other domains, including TQL (Thr, Gln, and Leu), GGPL (Gly, Gly, Pro, and Leu), and FFD (Phe, Phe, and Asp) (Cao et al., 2016).

The *GRF* gene family is a small family; therefore, the functions of each member of the GRF family in the studied species can be studied more comprehensively. Studies have found that *GRF* genes are often expressed strongly in actively growing and developing tissues, such as germinating seeds, ears, shoots, flower buds, and young leaves (Kim et al., 2003; Choi et al., 2004; Zhang et al., 2008; Wang et al., 2014; Zhang et al., 2017). In addition, studies have shown that most *GRF*s are regulated by miRNA396. For example, in *Arabidopsis*, seven miRNA396 target genes were predicted, and six *AtGRF*s were confirmed in the experiment (Jones-Rhoades and Bartel, 2004; Liu et al., 2009). With the completion of many plant genome sequences, *GRF* family members of some plants have been studied, such as *Arabidopsis* (Kim et al., 2003), rice (Choi et al., 2004), maize (Zhang et al., 2008), *Brachypodium distachyon* (Filiz et al., 2014), *Brassica rapa* (Wang et al., 2014), *Brassica napus* (Ma et al., 2017), *Solanu lycopersicum* (Khatun et al., 2017), *Nicotiana tabacum* (Zhang et al., 2017), Cucurbitaceae (Baloglu, 2014), *Manihot esculenta* (Shang et al., 2018), apple (Zheng et al., 2018), mulberry (Rukmangada et al., 2018), and so on. However, studies on *GRF* genes in foxtail millet and the evolutionary trajectory of *GRF* genes have not been available.

Foxtail millet is one of the oldest food crops in many regions of the world, especially in China and India, where it is still widely cultivated as a staple food. Although the genome of foxtail millet is small, it has a high inbreeding rate, strong C4 photosynthesis, and high nutritional value, which is usually higher than other grains, containing a large number of minerals, such as essential amino acids, carbohydrates, and vitamins (Li and Brutnell, 2011; Pandey et al., 2013; Jia et al., 2013; Ji et al., 2015; Li et al., 2018). With the sequencing and continuous updating of the foxtail millet genome, now the foxtail millet genome is about 515 Mb (Bennetzen et al., 2012; Zhang et al., 2012; Han et al., 2014; Yang et al., 2020). Together with other gramineous plants, foxtail millet was affected by a whole-genome duplication or tetraploidy approximately 100 million years ago (Wang et al., 2015). This event resulted in thousands of duplicated genes in the existing genome, providing evolutionary power for genetic and functional innovation. Studying *GRF*s in foxtail millet helps to improve crop genetics and contributes to in-depth study of *GRF* function and food production. In this study, we conducted a series of informatics analysis on the exploration and functional prediction of *GRF* using a more comprehensive bioinformatics method to lay the foundation for further study of *GRF* functions.

## Materials and Methods

### Acquisition of Members of the *GRF* Gene Family

We selected 20 plants (5 dicots, 7 monocots, 1 basal angiosperm, 1 Pteridophyta, 1 Bryophyta, and 5 green algae) for *GRF* evolution analysis, in which the genome-wide of *Aegilops tauschii* was obtained from the literature (Luo et al., 2017). The remaining 19 species were obtained from the JGI database (http://genome.jgi.doe.gov/) [*Arabidopsis thaliana Araport11*, *Carica papaya ASGPBv0.4*, *Populus trichocarpa v3.1*, *Vitis vinifera v2.1*, *Solanum lycopersicum ITAG3.2*, *Zea mays Ensembl-18*, *Sorghum bicolor Rio v2.1*, *Setaria italica v2.2*, *Brachypodium distachyon Bd21-3 v1.1*, *Hordeum vulgare r1*, *Oryza sativa v7.0*, *Amborella trichopoda v1.0*, *Selaginella moellendorfii v1.0*, *Physcomitrella patens v3.3*, *Chlamydomonas reinhardtii v5.6*, *Volvox carteri v2.1*, *Coccomyxa subellipsoidea C-169 v2.0*, *Micromonas* sp. *RCC299 v3.0,* and *Ostreococcus lucimarinus v2.0*]. We downloaded the WRC (PF08879) and QLQ (PF08880) domains from the Pfam database (Bateman et al., 2013). The HMMER (version 3.2.1) software (Mistry et al., 2013) was used to identify GRF candidate members in 20 species. In addition, we also used local BLAST to screen GRF family members of all species again. Finally, we used Pfam (http://pfam.xfam.org), CDD (https://www.ncbi.nlm.nih.gov/cdd), and SMART databases (http://smart.embl-heidelberg.de/) to confirm GRF members that contain WRC and QLQ domains.

### Phylogenetic Analysis of GRF Family

Full-length amino acid sequences of GRF in all species were aligned in MAFFT (version 7.037b) (Katoh and Standley, 2013) using auto strategy and were then manually adjusted in BioEdit (Hall, 1999). JTT + I + G + F model was determined to be the best model via ProtTest (version 3.4.2) (Darriba et al., 2011). PhyML 3.1 was used to construct ML trees with the above model and 1000 nonparametric bootstrap replicates (Guindon et al., 2010).

The amino acid sequences of 10 GRFs of foxtail millet were aligned by ClustalW (Thompson et al., 1994). We employed MEGA 7.0 to construct the phylogenetic trees of GRFs in foxtail millet by using the NJ method with the following parameters: Pairwise deletion and 1000 bootstrap replications (Kumar et al., 2016).

### Characterization of GRFs in Foxtail Millet

The chromosome distribution of *GRFs* in foxtail millet was drawn by MapChart software (Voorrips, 2002). The online website MEME (http://meme-suite.org/) was employed to analyze GRF proteins in foxtail millet to identify as the conservative motifs (Bailey et al., 2009). The maximum number of motifs was set to be 5, and the remaining parameters were default. Isoelectric point value and theoretical molecular weight of GRF proteins in foxtail millet were calculated using the ProtParam tool of ExPaSy (https://web.expasy.org/protparam/) (Gasteiger et al., 2005). The subcellular localization of GRFs in foxtail millet was predicted by Plant-mPLoc database (Chou and Shen, 2010). Using the SOPMA website (https://npsa-prabi.ibcp.fr/cgi-bin/npsa_automat.pl?page=npsa_sopma.html) to predict the secondary structure of GRF proteins in foxtail millet, the parameters were default. Using the Phyre2 website (http://www.sbg.bio.ic.ac.uk/∼phyre2/html/page.cgi?id=index) to predict the three-dimensional structure of GRF proteins, the parameters were default. The gene structure of GRFs in foxtail millet was analyzed and drawn using GSDS 2.0 (http://gsds.cbi.pku.edu.cn/) (Hu et al., 2014) and CFVisual (version 2.1) (https://github.com/ChenHuilong1223/CFVisual) (Chen et al., 2021). Multiple sequence alignment of the amino acid sequences was performed by ClustalX (Wilm et al., 2007) and conserved regions were visualized using DNAMAN 8.0. In order to reduce errors, nine coding sequences that meet requirements were screened, based on literature criteria (Eyre-Walker, 1991). Afterward, the codon bias analysis of these sequences was performed via CodonW software (https://sourceforge.net/projects/codonw/).

### Selection Pressure, Gene Duplication, and Collinearity Analysis

The amino acid sequences of GRFs in foxtail millet were aligned using MAFFT (version 7.037b), and the amino acid alignments were translated into coding sequence alignments via PAL2NAL (http://www.bork.embl.de/pal2nal/) (Suyama et al., 2006). After that, we employed the codeml program in PAML 4.9 h software (Yang, 1997) to calculate the selection pressure of each branch of the GRF phylogenetic tree. We chose the branch model to achieve this (Yang et al., 1998), which was based on the free ratio model and one ratio model (prob = 1.517e-04).

MCScanX software (Wang et al., 2012) was used to analyze the duplications of the GRF family of foxtail millet, and 34,584 protein sequences from foxtail millet (Bennetzen et al., 2012) were analyzed using all-vs-all BLAST search with e-value < 1e-05 (Camacho et al., 2009). The putative WGDs/segmental duplications of *GRF* genes located on chromosomes of foxtail millet are connected by red lines. *Ks* (synonymous substitution rate) and *Ka* (nonsynonymous substitution rate) values of WGDs/segmental duplications were calculated based on the coding sequence alignments using the method of Nei and Gojobori as implemented in KaKs_calculator 2.0 (Nei and Gojobori, 1986; Wang et al., 2010). The *Ks* value was translated into duplication time in millions of years based on the rate of λ substitutions per synonymous site per year. The duplication time of duplicated genes was calculated by *T* = *Ks*/2 λ × 10^–6^ Mya ( λ = 6.5 × 10^–9^ for grasses) (Lynch and Conery, 2000; Mehanathan et al., 2014; Wang et al., 2015; Chai et al., 2018). To reduce errors, we only analyzed the results for *Ks* < 1.

Orthologous pairs of *GRF* members among foxtail millet, *Arabidopsis*, and rice were identified using OrthoFinder software (version 2.2.6) (Emms and Kelly, 2015) and OrthoMCL (version 2.0.9) (Li et al., 2003). The results were visualized using Circos (version 0.69–6) (Krzywinski et al., 2009).

### Expression and Regulation Analysis of *GRF*s in Foxtail Millet

We obtained the sequence of foxtail millet miRNA396 from the literature (Yadav et al., 2016), and then used psRNATargetsoftware (http://plantgrn.noble.org/psRNATarget/) to predict the binding site of miR396 in *GRF* genes of foxtail millet (Dai et al., 2018). PlantCARE (http://bioinformatics.psb.ugent.be/webtools/plantcare/html/) was used to analyze the 1 Kb sequence upstream of *GRF* genes in foxtail millet (Lescot et al., 2002). We utilized an in-house Python script to extract *GRF* TPM values of foxtail millet from Illumina RNA-seq data reported previously (Yang et al., 2020). The heatmap was drawn via Morpheus software (https://software.broadinstitute.org/morpheus/) based on the transformed data of log2 (TPM+1) values. The String database (version 11.0) (https://string-db.org/) was used to predict interaction proteins of GRFs with the minimum required interaction score set to be high confidence (0.700) (Szklarczyk et al., 2015). The agriGO V2.0 was used for GO analysis of GRFs in foxtail millet (Tian et al., 2017).

### Quantitative Real-Time PCR Analysis

The total RNA was extracted using RNAprep Pure Plant Plus Kit (TIANGEN) from three tissues: imbibed 3-day seed, 2-week-old seedling, and 2-week-old seedling root. First-strand cDNA was synthesized using Fastking RT Kit (with gDNase) (TIANGEN). The SuperReal PreMix Plus (SYBR Green) (TIANGEN) was used for real-time-qPCR analysis with 7900HT Fast Real-Time PCR System (American Applied Biosystems). The Primers were designed by Primer Premier6.0 and synthesized by GENEWIZ Biotechnology Co., Ltd. (Supplementary Table S1). EF-1α was the reference gene (Kumar et al., 2013).

## Results

### Genome-wide Identification and Classification of *GRF* Genes in Plants

**FIGURE 1:**
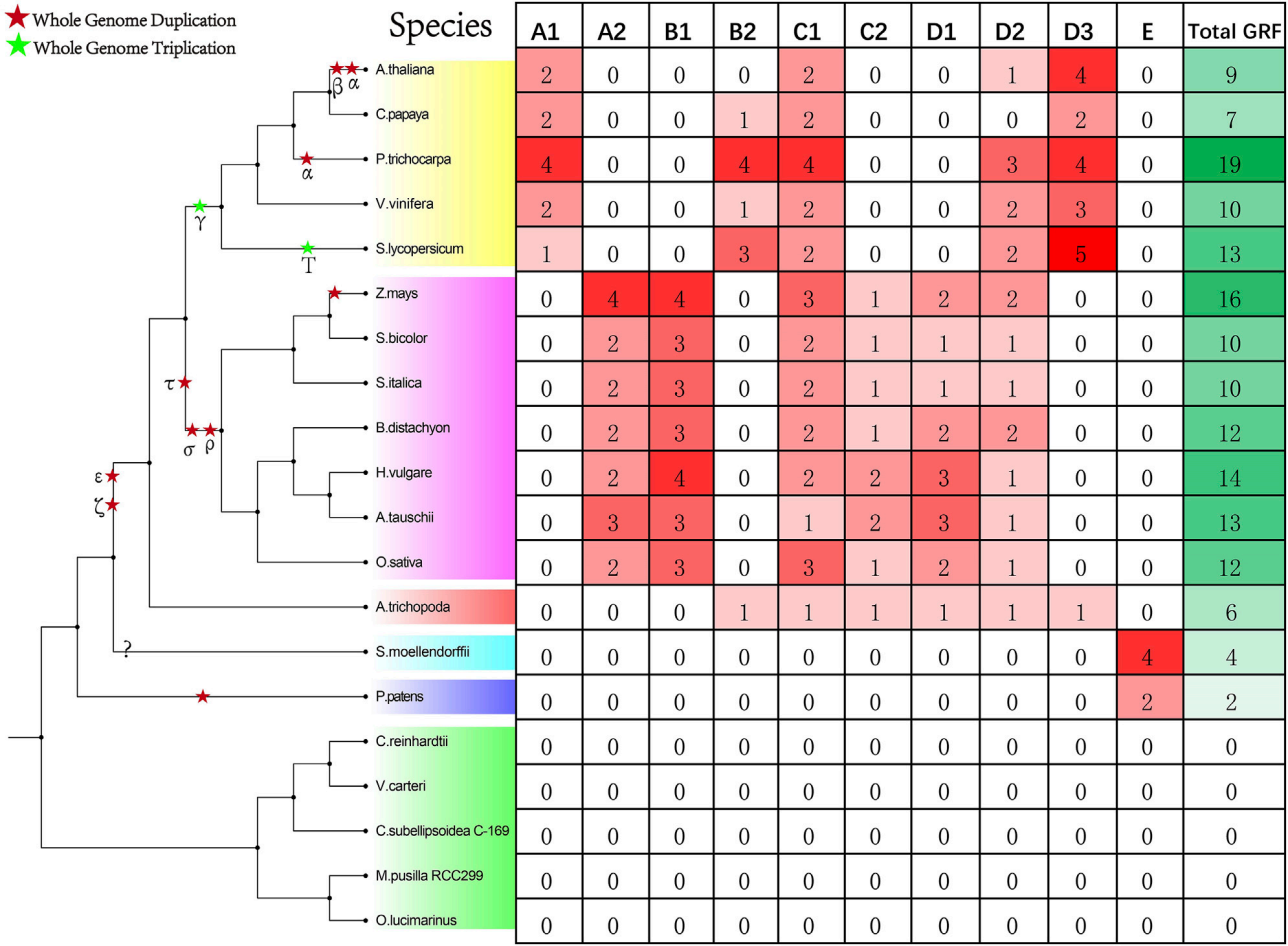
The number of *GRF* families in the collected species. The left of this figure shows the evolutionary relationships of the species; the right of this figure shows the number detail of the *GRF* family of each species.

We identified a total of 157 *GRF* genes in 20 species (Figure 1, Supplementary Table S2). No *GRF* gene has been identified in green algae (*C.reinhardtii*, *V. carteri*, *C. subellipsoidea C-169*, *M.* sp. *RCC299,* and *O. lucimarinus*). In land plants, the least *GRF* genes (two) have been identified in *P. patens*, four *GRF* genes have been identified in *S. moellendorfii,* and six *GRF* genes have been identified in *A. trichopoda*. The number of *GRF*s in monocots (*Z.mays*, *S. bicolor*, *S. italica*, *B. distachyon*, *H. vulgare,* and *O. sativa*) ranges from 10 to 16, while the number of *GRF*s in dicots (*A.thaliana*, *C. papaya*, *P. trichocarpa*, *V. vinifera,* and *S. lycopersicum*) ranges from 7 to 19.

**FIGURE 2:**
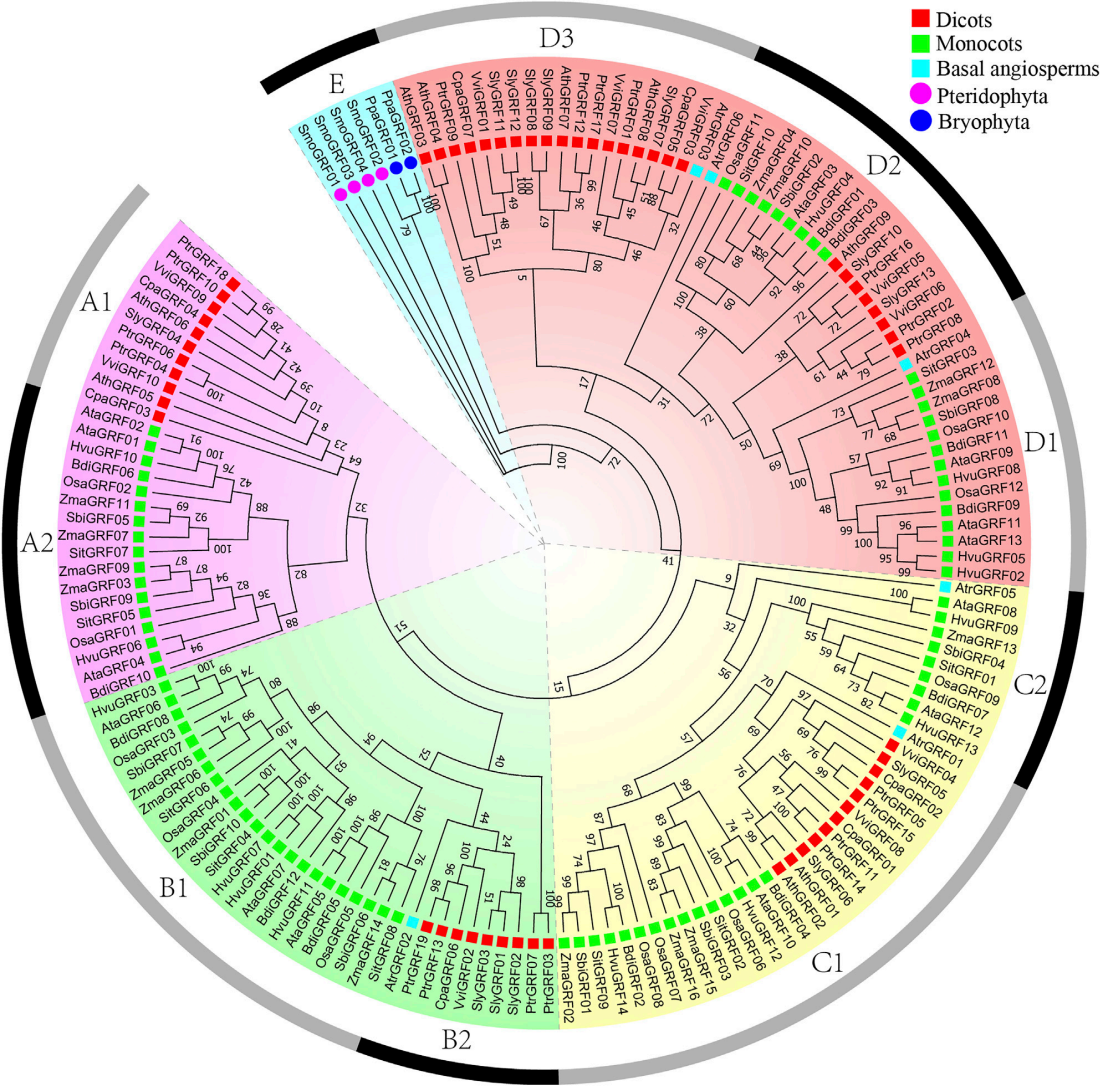
Phylogenetic trees of *GRF* genes. Phylogenetic analysis of plant *GRF* proteins using PhyML 3.1 under JTT + I + G + F model with 1000 nonparametric bootstrap replicates.

According to previous research and the phylogenetic tree topology (Song et al., 2018), phylogenetic analysis showed that 157 *GRF* genes could be clustered into five categories: A, B, C, D, and E (Figure 2). A class is subdivided into A1 and A2 subclass, and B class is subdivided into B1 and B2 subclass. C class is subdivided into C1 and C2 subclass, and D class is subdivided into D1, D2, and D3 subclass. According to statistics, the genes of the A1 subclass are dicots *GRF*s. The genes of the A2 subclass and B1 subclass are monocots *GRF*s. The genes of the B2 subclass and D3 subclass contain some dicots *GRF*s and one basal angiosperm *GRF*. The genes of the C2 subclass and D1 subclass contain some monocots *GRF*s and one basal angiosperm *GRF*. In addition, the genes of ancient E class are *GRF*s of all ancient species (*P. patens* and *S. moellendorfii*) (Figure 1).

### Gain and Loss of *GRF* Genes in Plants

**FIGURE 3:**
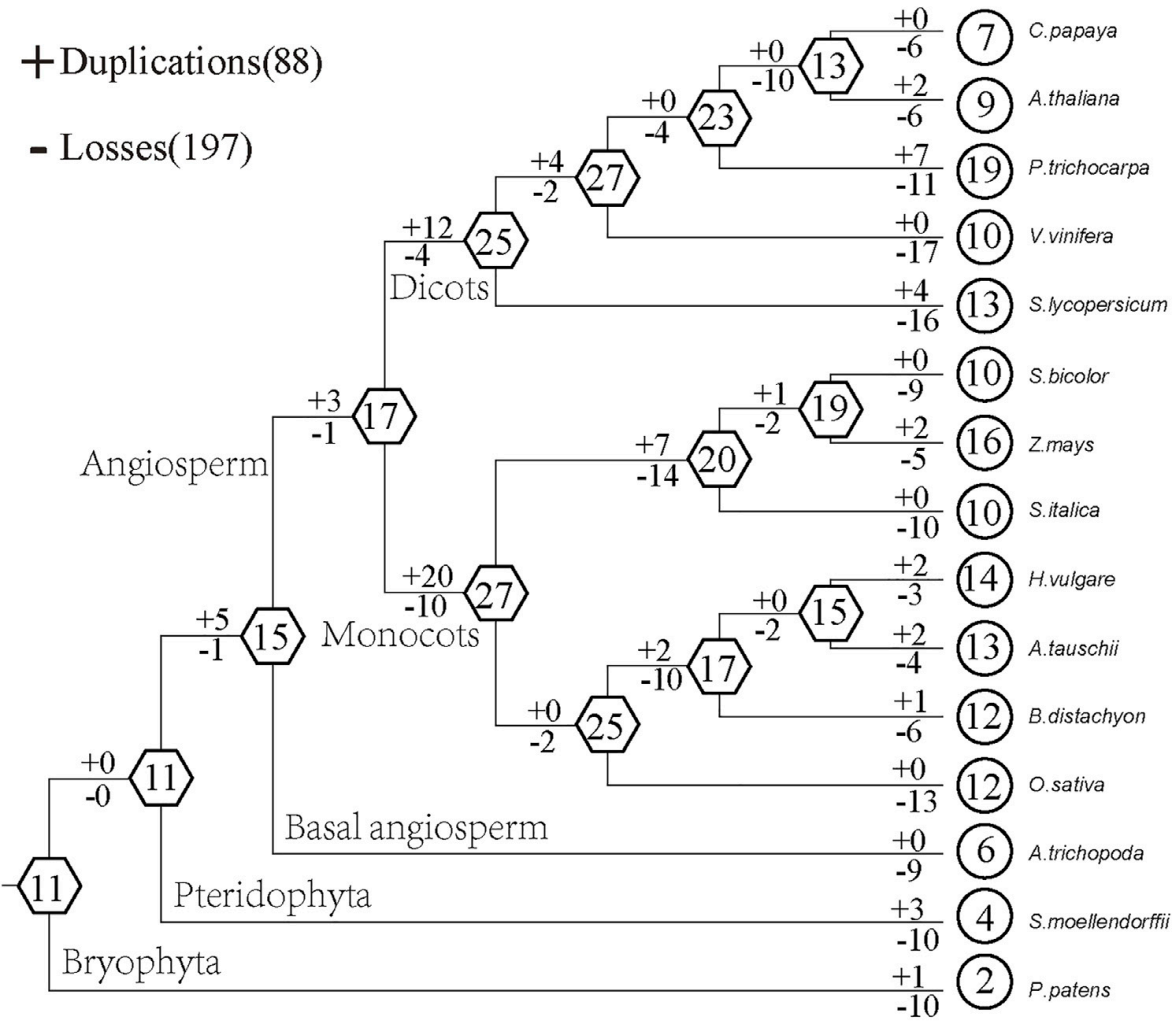
Schematic diagram of gain and loss of *GRF* gene family in plants. The numbers in the hexagons and circles represent the number of *GRF* genes in ancestors and existing species, and the + and − signs represent the gain and loss of genes, respectively.

Based on the comparison between the species tree and the plant *GRF* gene tree, we used Notung software to analyze the gain and loss of *GRF* genes. The results show that the ancestors of land plants contained 11 *GRF* genes (Figure 3). The loss is more serious in lower plants. Among them, 10 *GRF* genes of *P. patens* and *S. moellendorfii* have been lost, and 1 *GRF* gene and 3 *GRF* genes have been obtained, respectively. The ancestor of angiosperms contains 15 *GRF* genes with 9 *GRF* genes being lost and 0 *GRF* genes being gained. This results in 6 existing *GRF* genes in *A. trichopoda*. There are 17 *GRF* genes in common ancestors of dicots and monocots. After 12 *GRF* genes were gained and 4 *GRF* genes were lost, 25 *GRF* genes exist in dicots ancestor species, after 20 *GRF* genes were gained and 10 *GRF* genes were lost, 25 *GRF* genes exist in monocots ancestor species. This indicates that the *GRF* gene family in the ancestor species of angiosperms has expanded after the divergence of monocots and dicots. After comparing the gain and loss of dicots with that of monocots, we found that the *GRF* gene family in monocots was gradually shrinking. For example, the ancestor of foxtail millet, sorghum, and maize has undergone 14 losses and 7 gains, which result in the reduction from 27 *GRF* genes to 20 *GRF* genes. Although the number of existing species of dicots is less than that of ancestors, it does not show a gradual shrinking phenomenon. For example, the ancestor of *V. vinifera*, *P. pilosa*, *A. thaliana,* and *C. papaya* has undergone 2 losses and 4 gains, but the number of *GRF* genes of the ancestor increased from 25 to 27.

### Strong Collinearity Between Foxtail Millet *GRFs* and Related Species and Weaker Positive Selection

Combining with the results of internal collinearity in foxtail millet, we found that 6 pairs of *GRF* genes (seven genes, accounting for 70.00%) are in the collinearity block. Estimates of divergence time indicate that the divergence time of fragment duplication ranges from 15.07 Mya to 45.97 Mya, and *GRF*s are subject to purification options (Supplementary Table S3). Unfortunately, we did not find a tandem repeat gene pair.

**FIGURE 4:**
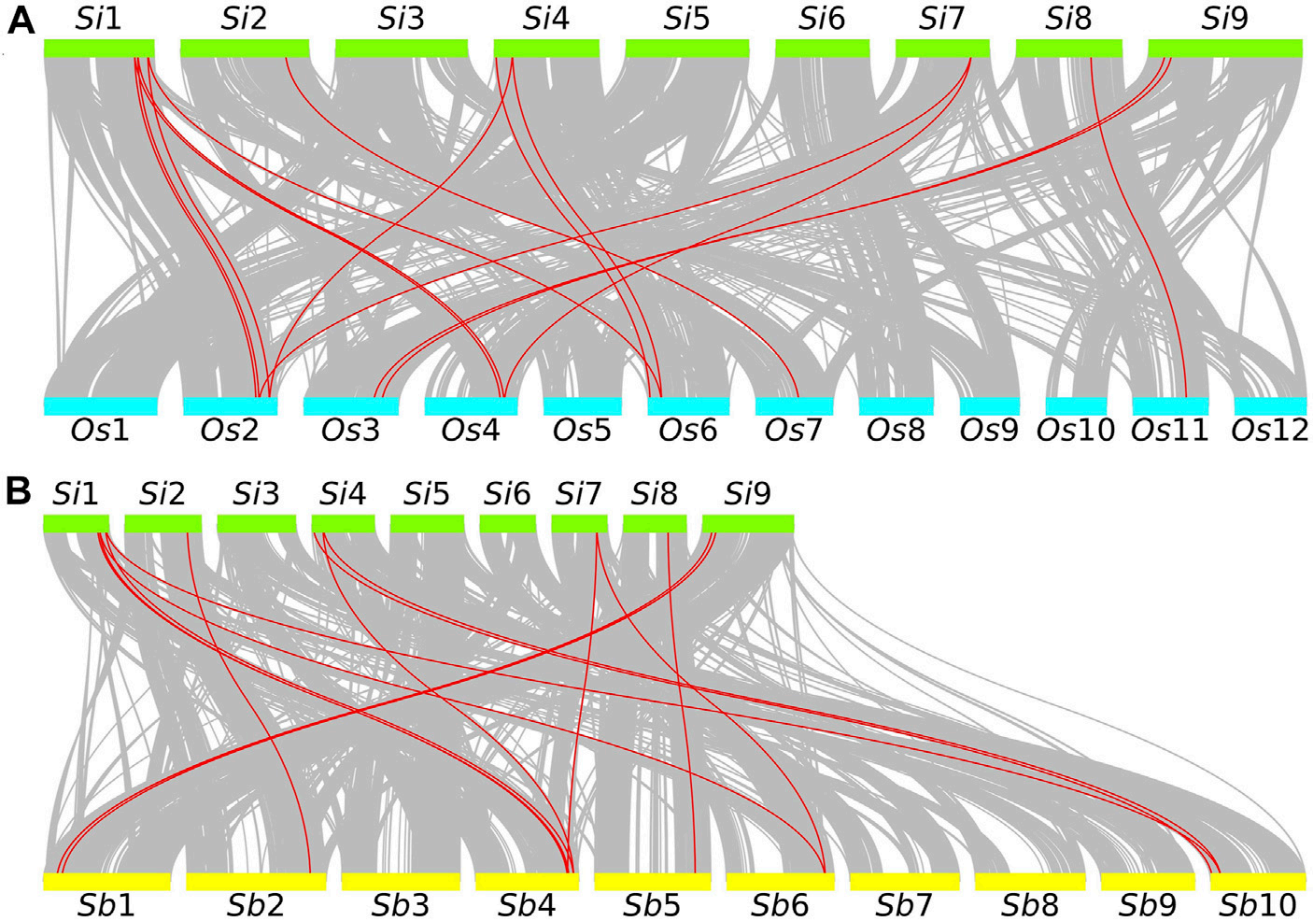
Collinearity analysis of foxtail millet *GRF* and related species. **(A)** The green rectangular color block represents the foxtail millet chromosome. *Si* represents foxtail millet. The number represents the chromosome number. The blue rectangular color block represents the rice chromosome. *Os* represents rice. The number represents the chromosome number. **(B)** The green rectangular color block represents the foxtail millet chromosome. *Si* represents foxtail millet. The number represents the chromosome number. The yellow rectangle represents the sorghum chromosome. *Sb* represents sorghum. The number represents the chromosome number.

In addition, we used a self-made Python script (https://github.com/ChenHuilong1223) to draw the *GRF* collinearity relationship between foxtail millet and other closely related species. We identified 15 pairs of collinearity genes in the collinearity region of the genome of foxtail millet and rice. Chromosome 1 of foxtail millet has the most collinearity *GRF* gene pairs (40.00%) with rice. Among them, there are collinearity *GRF* genes with chromosome 2, 4, and 6 of rice, respectively (Figure 4A). Similarly, 15 collinearity gene pairs (33.33%) were identified in foxtail millet and sorghum. Chromosome 1 of foxtail millet has the most collinearity *GRF* gene pairs with sorghum. Among them, there are collinearity *GRF* genes with chromosome 4, 6, and 10 of sorghum (Figure 4B). An abundance of collinearity gene pairs indicate that *GRF* of foxtail millet has strong collinearity with closely related species.

**FIGURE 5:**
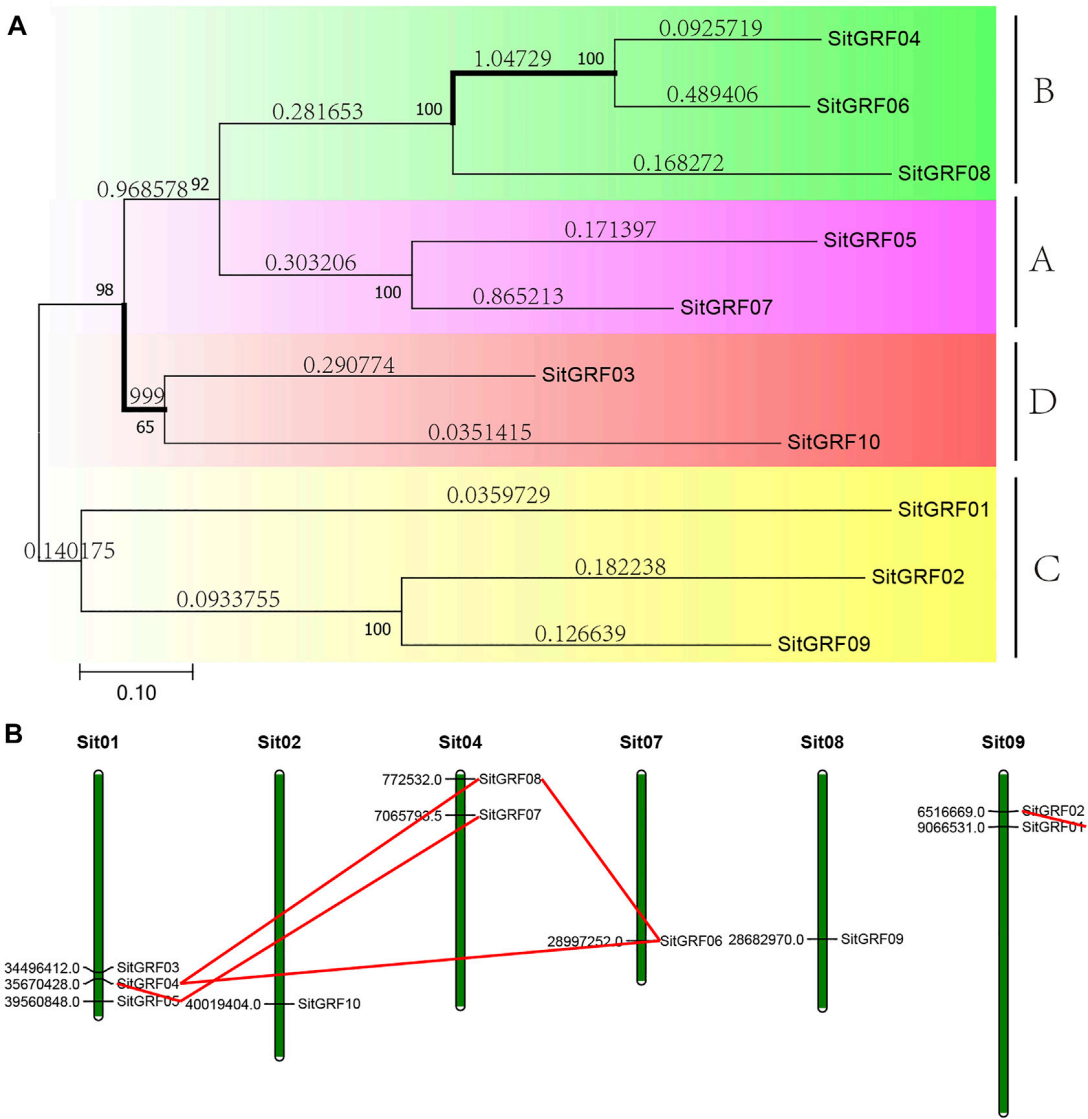
Phylogenetic tree and chromosome location of the GRF family of foxtail millet. **(A)** Phylogenetic analysis of foxtail millet GRF proteins using MEGA 7.0 via the neighbor-joining (NJ) method with 1000 bootstrap replicates. Bold branches suggest that they may be positively selected. **(B)** Chromosomal distribution and gene duplications of foxtail millet *GRF* genes. Green bars represent chromosomes of foxtail millet. The putative whole genome duplication (WGD) or segmental duplication genes are linked by a red line.

The *GRF* phylogeny tree of foxtail millet shows that these 10 *GRF* genes can be assigned to A, B, C, and D class in the phylogenetic tree (Figure 5A). After selection pressure analysis, two (11.76%) of the 17 branches in the *GRF* gene tree of foxtail millet were detected to be positively selected. Therefore, this indicates that the *GRF* of foxtail millet has received weaker positive selection during the evolution process.

### Characterization and Structure of *GRF*s in Foxtail Millet

We have identified 10 *GRF* genes in the foxtail millet genome. The amino acid lengths of GRFs in foxtail millet are between 232 and 590 amino acids. The molecular weights (MW) are between 34,981.10 and 61,607.68 Da. The predicted isoelectric point (PI) values are between 4.95 and 9.54. Prediction of subcellular location indicates that all 10 GRFs may be located in the nucleus (Supplementary Table S4).

The chromosome location shows that there is no *GRF* gene on chromosomes 3, 5, and 6 of the foxtail millet, no clustering phenomenon and scattered distribution on those chromosomes (Figure 5B). The remaining chromosomes have one to three *GRF* genes with the most being on chromosome 1.

**FIGURE 6:**
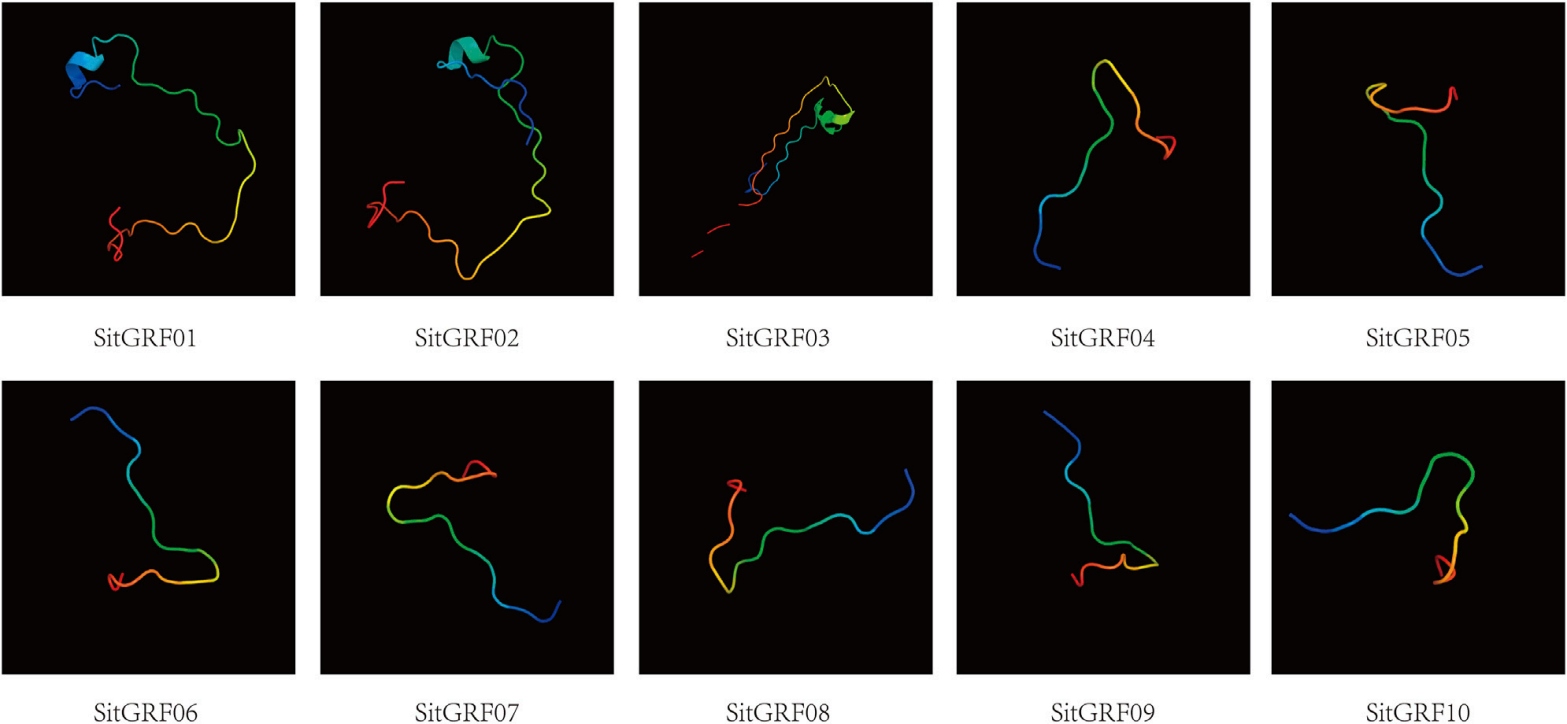
Three-dimensional structures of GRF proteins in foxtail millet.

Regarding the composition of the secondary structure of GRF in foxtail millet, a random coil occupies the largest proportion (49.63–67.83%). Alpha helix occupies the second largest proportion (18.26–36.57%), and extended strand occupies the third largest proportion (4.56–12.03%). Beta turn makes up the smallest proportion (3.04–6.08%) (Supplementary Table S5). In addition, we predicted the three-dimensional structure of the GRFs in foxtail millet. The results showed that the three-dimensional structure of the GRFs in foxtail millet is simple with no complicated spiral folding structures. The three-dimensional structure of the 10 GRFs is very similar (Figure 6).

**FIGURE 7:**
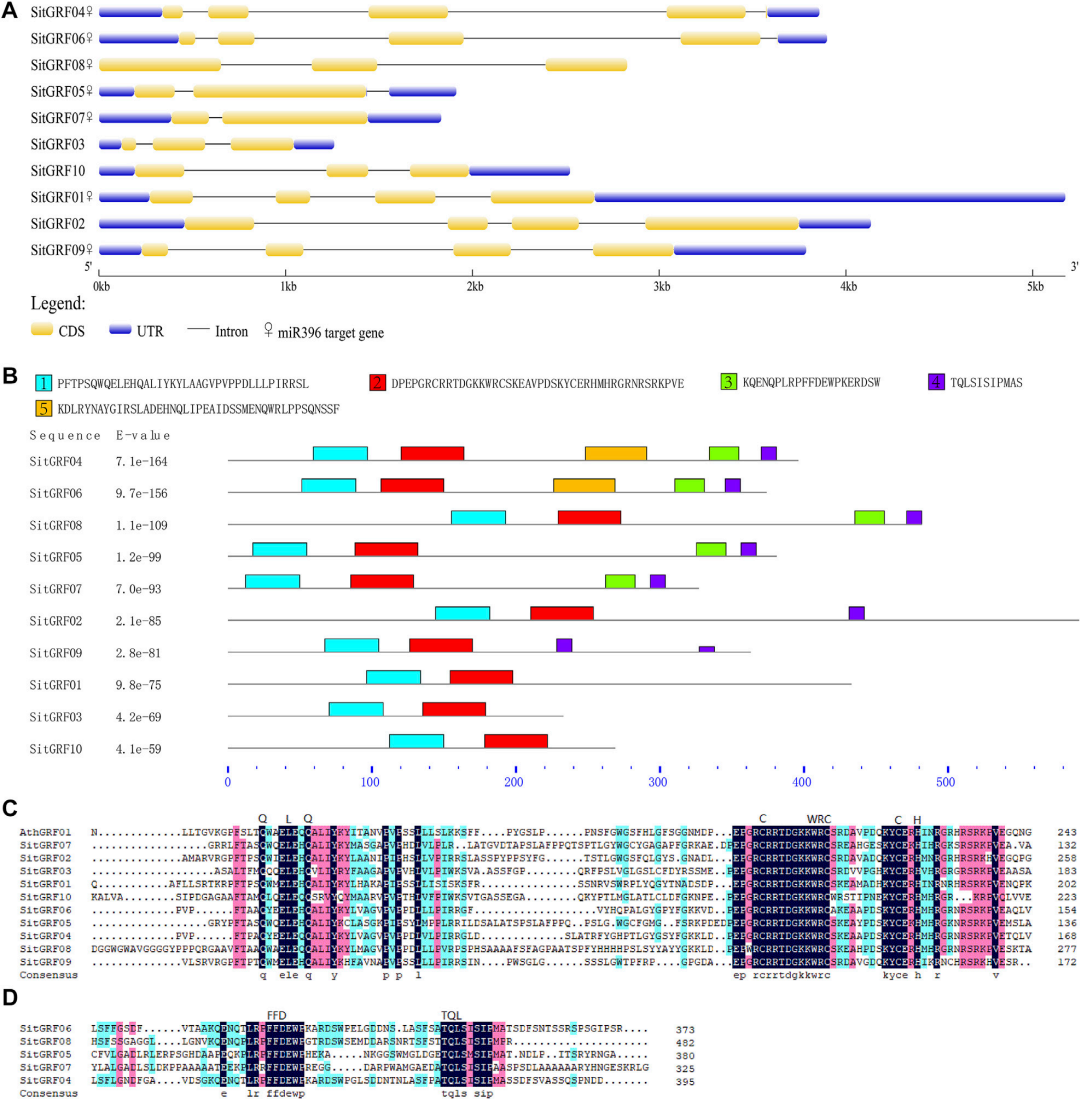
Characterization of foxtail millet *GRF*s. **(A)** Exon-intron organization and miR396 binding information of foxtail millet *GRF* family. **(B)** Distributions of conserved motifs in foxtail millet *GRF* genes. Five putative motifs are indicated in different colored boxes. **(C)** Comparison of the amino acid sequences of foxtail millet GRF QLQ and WRC domains. **(D)** Multiple sequence alignment of the amino acid sequences of foxtail millet GRF other domains.

The results of the amino acid sequence alignment of GRFs in foxtail millet indicate that all GRFs in foxtail millet contain QLQ and WRC (Figure 7C). The WRC domain contains a C3H motif spanning three cysteines and one histidine. The motif is CX9CX10CX2H, and the motif of the QLQ domain is QX3LX2Q. We also found that five GRF proteins contain FFD and TQL domains and are highly conserved (Figure 7D).

We used MEME software to predict the GRF motifs in foxtail millet (Figure 7B). The results indicated that the conservative motifs 1 through 4 correspond to the corresponding domains. For example, motif 1 corresponds to QLQ, and motif 2 corresponds to WRC. In addition, we predicted that motif 5 exists in most members (seven) of the GRF family in foxtail millet.

In the structure of *GRF*s in foxtail millet, each *GRF* has two to four coding sequences (Figure 7A). The number of introns varies from one to four. It is noteworthy that *SitGRF08* has no UTR, and the 3′ UTR of *SitGRF01* is particularly long. However, *SitGRF03* has the shortest length of the gene structure.

We also conducted the analysis of codon preference, and the results showed that the *GRF* gene family of foxtail millet is biased toward the use of G or C nucleotides. The ENC value is between 32.68 and 61.00, and 35.00 is the strength of codon preference. The closer to 61.00, the weaker the codon preference is, and vice versa. It shows that the overall preference of *GRF*s in foxtail millet is relatively weak (Supplementary Table S6). We also identified 19 optimal codons: UUU, UUG, AUU, GUA, UCU, CCG, ACG, GCA, UAA, CAU, CAG, AAU, AAG, GAU, GAA, UGC, CGC, AGU, and GGG (Supplementary Table S7). These results are helpful in the application of transgenic technology on foxtail millet.

### *cis*-acting Elements and miRNA

**FIGURE 8:**
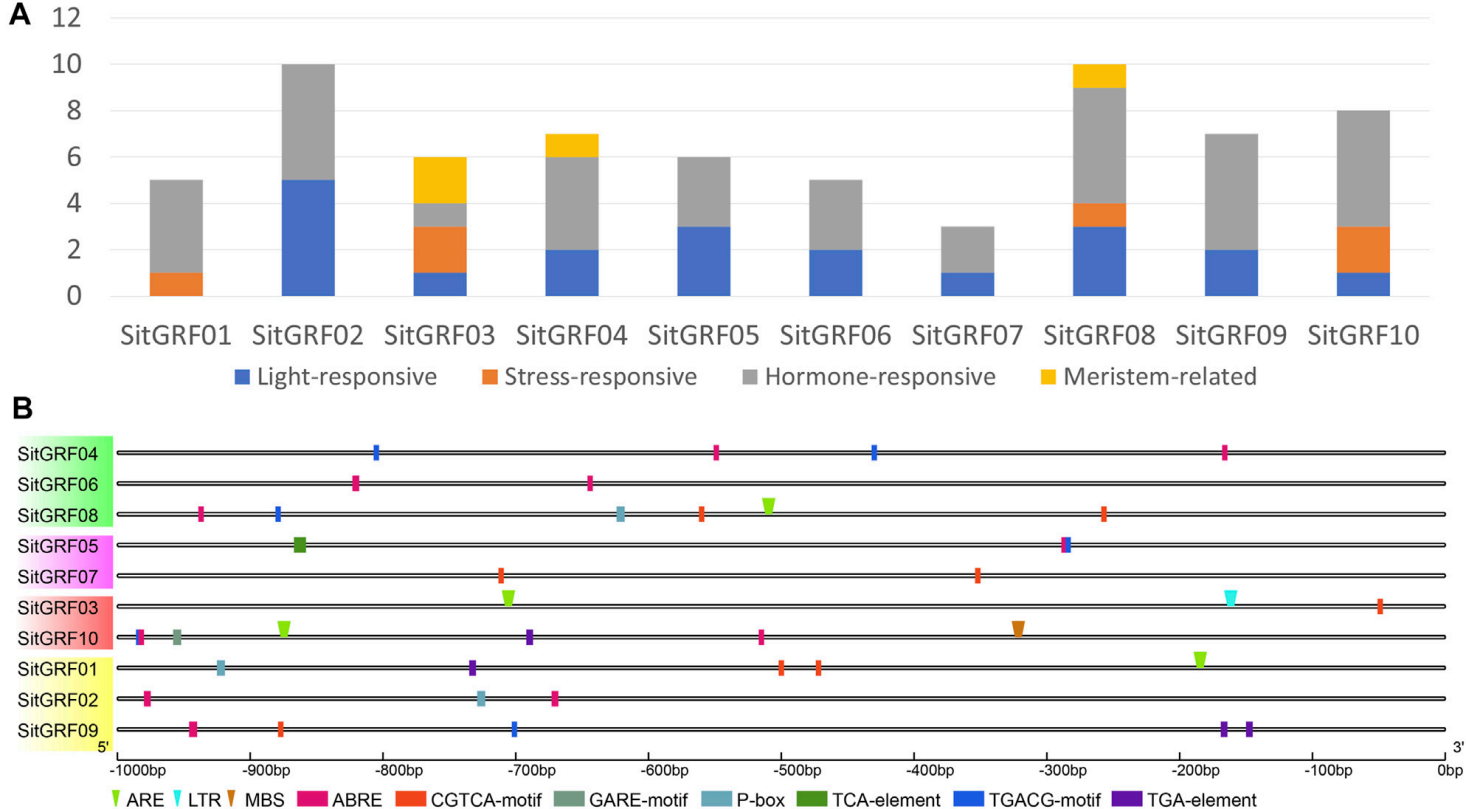
Promoter analysis of foxtail millet *GRF* genes. **(A)** Distribution of four kinds of *cis*-regulatory elements in the promoter of foxtail millet *GRF* genes. **(B)** The 1 Kb promoter sequences of corresponding *GRF* genes were used to analyze hormone-related *cis*-elements and stress-related elements. Rectangle indicates stress-related elements, and the inverted triangle indicates hormone-related elements. Different *cis*-elements were indicated by different colored symbols and placed in their relative position on the promoter.

We predicted *cis*-acting elements of the putative promoter region (upstream 1000 bp) of each *GRF* in foxtail millet (Figures 8A,B, Supplementary Table S8). We found that all gene promoters contained hormone-related *cis*-acting elements, and only four gene promoters were predicted to contain stress-related *cis*-acting elements. Seven hormone-related *cis*-acting elements were identified in the promoter region of *GRF*s in foxtail millet. These seven hormone-related *cis*-acting elements are ABRE (*cis*-acting element involved in abscisic acid responsiveness) (Hobo et al., 1999), CGTCA/TGACG-motif (*cis*-acting element involved in MeJA-responsiveness) (Rouster et al., 1997), GARE-motif/P-box (gibberellin-responsive *cis*-acting element) (Gubler and Jacobsen, 1992), TCA-element (*cis*-acting element involved in salicylic acid responsiveness) (Shah and Klessig, 1996), and TGA-element (auxin-responsive *cis*-acting element) (Khan et al., 2012). Three stress-related *cis*-acting elements were identified in the promoter region of *GRF*s in foxtail millet. The three stress-related *cis*-acting elements are ARE (*cis*-acting element essential for anaerobic induction), LTR (*cis*-acting element involved in low-temperature responsiveness), and MBS (MYB binding site involved in drought-inducibility) (Yoshida et al., 1998).

According to previous studies, most *GRF*s are regulated by miR396 (Jones-Rhoades and Bartel, 2004; Liu et al., 2009; Zheng et al., 2018). Therefore, we predicted the *GRF* target gene of Sit-miR396 (Figure 7A, Supplementary Table S9). The results show that seven *GRF* target genes are predicted, and they all have a cleavage effect on each *GRF* gene.

### Tissue Expression Analysis of *GRF* Genes in Foxtail Millet at Different Periods

**FIGURE 9:**
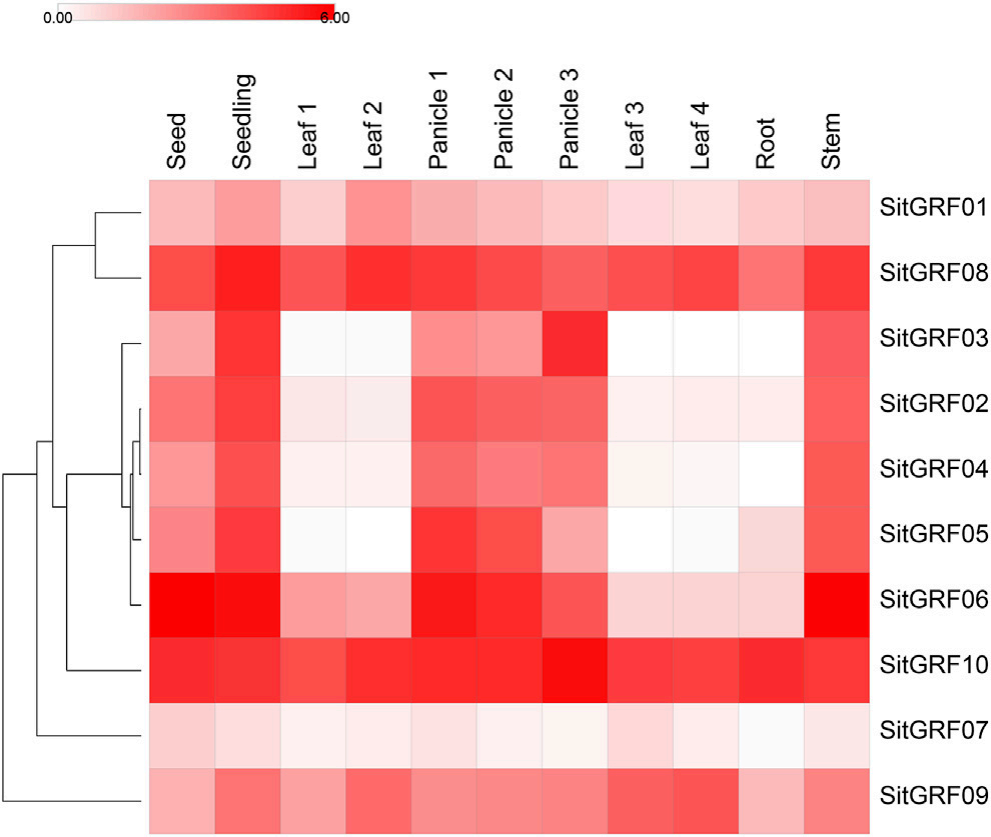
Expression of foxtail millet *GRF* genes in the different organs and periods. The shade of the color corresponds to the expression value (log2 (TPM+1)). Seed represents 3 days imbibed seeds; seedling represents 2-week-old whole seedling. Leaf 1 represents the top first fully extended leaf of a 2-week-old seedling. Leaf 2 represents the top second leaf of 30-day-old plants. Panicle 1 represents an immature panicle. Panicle 2 represents a panicle at the pollination stage. Panicle 3 represents a panicleat at the grain-filling stage. Leaf 3 represents a flag leaf. Leaf 4 represents the fourth leaf. Root represents the root. Stem represents the stem.

By analyzing the expression pattern of *GRF* genes in foxtail millet in different tissues (Figure 9, Supplementary Table S10), the results showed that the expression of *GRF*s in foxtail millet was the strongest in seed, panicle, and stem tissues. The weakest expression patterns of *GRF* genes in foxtail millet were in different periods and were found during the third day of imbibed seeds. Some genes displayed higher expression, such as *SitGRF06* (68.12 TPM) and *SitGRF10* (29.86 TPM), followed by *SitGRF08* (16.66 TPM). In the seedling stage on the 14th day, the expression of seven *GRF* genes (70.00%) all increased, while *SitGRF06*, *SitGRF10,* and *SitGRF07* decreased. When the top first leaf of a 2-week-old seedling is fully extended, the gene expression changed little while the expression of *SitGRF01*, *SitGRF09*, *SitGRF08,* and *SitGRF10* increased. In immature panicles, *SitGRF02*, *SitGRF03*, *SitGRF04*, *SitGRF05,* and *SitGRF06* were significantly increased. The overall performance of the *GRF* family decreased gradually in the panicle at the pollination stage and at the grain-filling stage. However, *SitGRF03* and *SitGRF10* increased in the panicle at the grain-filling stage. In the flag leaf and the fourth leaf, the expression of seven *GRF* genes was extremely low while *SitGRF07*, *SitGRF08,* and *SitGRF10* had higher expression. Additionally, the overall expression of the *GRF* family was extremely low and nine *GRF* genes (except *SitGRF07*) had high expression values in the stem tissue. Through observation, we found that *SitGRF08* and *SitGRF10* maintained high expression in the organization of each period, and *SitGRF07* expressed lower in the organization of each period.

**FIGURE 10:**
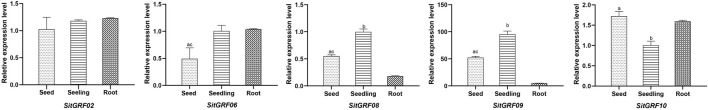
Tissue specific expression analysis of *SitGRF* genes in germinated seed, 2-week-old seedling, and root. The bars represent the mean values of three replicates ± s.d. Significant differences in means are indicated by a, b, c, *p* < 0.01, according to one-way ANOVA test. a represents the comparation between germinated seed and seedling. b represents the comparation between seedling and root. c represents the comparation between germinated seed and root.

In order to definitely test the tissue expression patterns of *SitGRF* genes, qRT-PCR was used to detect the relative expression level of five *SitGRF* genes (*SitGRF02*, *SitGRF06*, *SitGRF08*, *SitGRF09,* and *SitGRF10*) in imbibed 3-day seed, 2-week-old seedling, and the root of 2-week-old seedling. The other five *SitGRF* genes were not detected due to the lack of screening specific primers across the introns. The results of qRT-PCR showed that among 15 pairs of comparison (five genes and three tissues), the expression trend of 13 pairs (86.67%) was consistent with the transcriptome data (Figure 10). For example, among the three tissues, *SitGRF08* and *SitGRF09* expressed the highest in the seedling and the lowest in the root, *SitGRF10* expressed the highest in the germinated seed and the lowest in the seedling, In the meanwhile, both *SitGRF02* and *SitGRF06* expressed higher in the seedling than in the germinated seed. Only the expression pattern in the root of *SitGRF02* and *SitGRF06* was inconsistent. In qRT-PCR, *SitGRF02* and *SitGRF06* expressed the highest in the root, while in transcriptome, they were the lowest in the root.

### Protein Interaction Analysis

**FIGURE 11:**
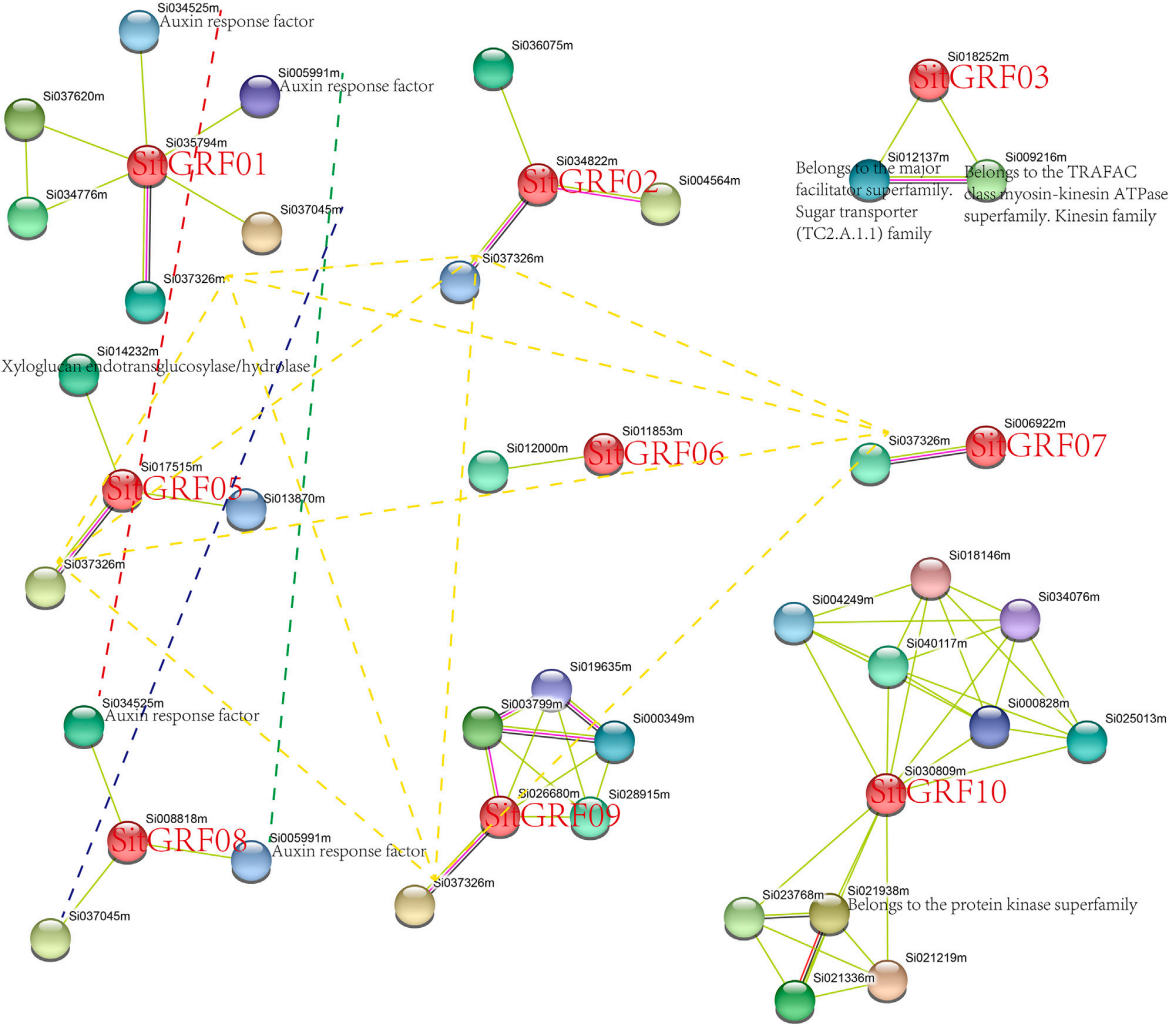
Protein interaction analysis of foxtail millet GRFs. The same protein is connected by a dotted line. The unannotated protein indicates uncharacterized protein in the String database.

In order to further explore the mechanism of action of proteins expressed by the *GRF* genes of foxtail millet, we looked for an interaction protein for each GRF in the String database (Figure 11). Under high confidence (0.7) conditions, GRFs in foxtail millet have interaction proteins, except SitGRF04. Among them, SitGRF10 had the most interaction proteins. Each of SitGRF06 and SitGRF07 has only one interaction protein. These interaction proteins provide clues to the function and mechanism of each *GRF*. For example, both SitGRF01 and SitGRF08 could interact with auxin response factors, implying that they may participate in the network regulation mechanism of auxin response factors. We also found that SitGRF01, SitGRF02, SitGRF05, SitGRF07, SitGRF08, and SitGRF09 can interact with Si037326m.

### Identification of Orthologs with Model Plants and Functional Annotation of GO

**FIGURE 12:**
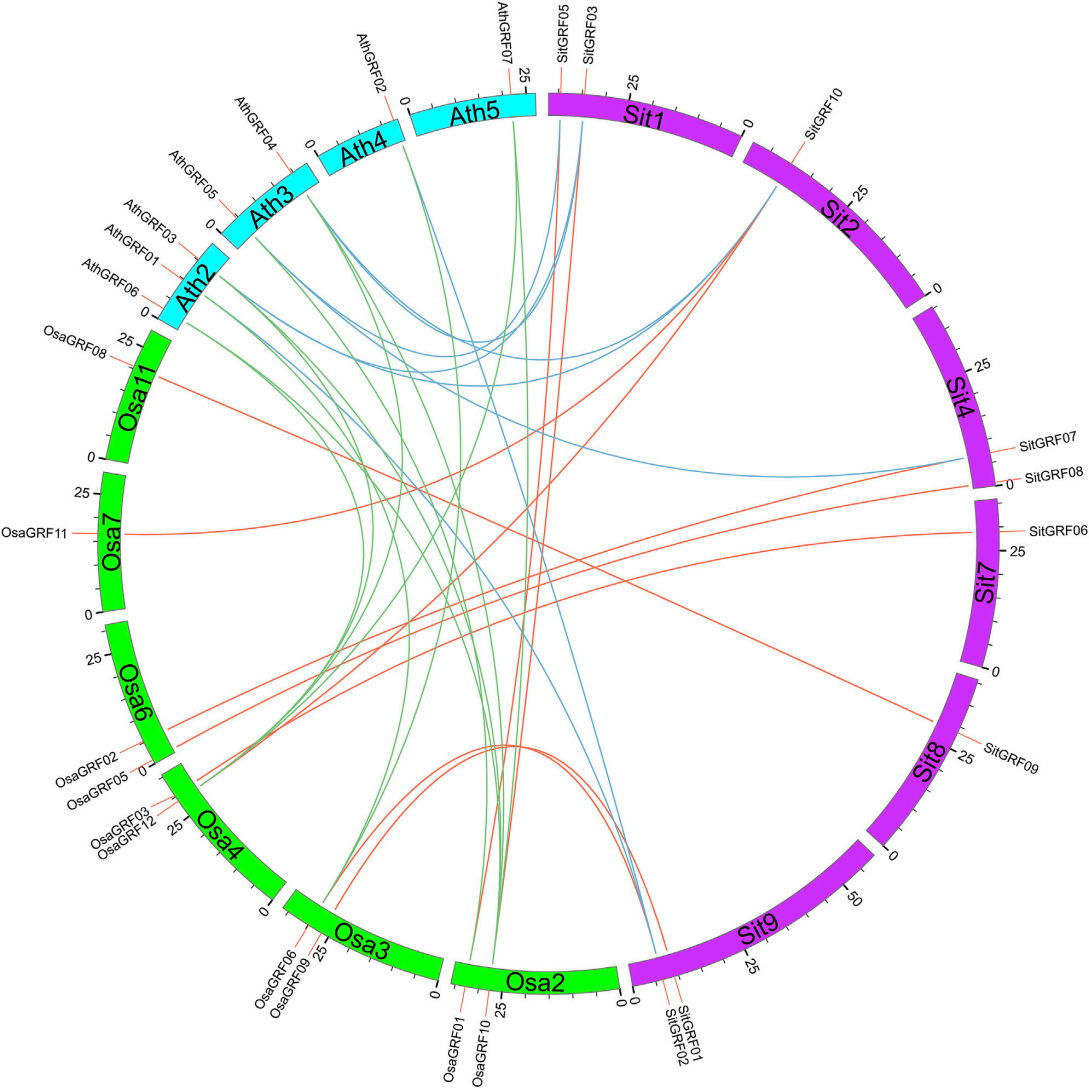
Orthologs of *GRF* genes between foxtail millet and model plants. The orthologous *GRF* gene pairs between different species are linked by different colors.

The identification of orthologous genes with *GRF* genes in model plants is helpful for the function prediction of *GRF* genes in foxtail millet. Through the identification of orthologous gene pairs of *GRF* with rice and *Arabidopsis*, it is found that the *GRF* gene family of foxtail millet and rice can form 10 pairs of orthologous genes. the *GRF* gene family of foxtail millet and rice have eight orthologous gene pairs. In addition, there are 10 orthologous gene pairs of the *GRF* gene family between *Arabidopsis* and rice (Figure 12).

We performed GO function annotations and the results reported that all 10 GRFs of foxtail millet can participate in biological pathways, molecular functions, and cellular components (Supplementary Table S11). A total of 75 GO numbers could be annotated. The most annotated entries are in biological pathways (64.00%), such as developmental process, regulation of cellular biosynthetic process, regulation of cellular metabolic process, etc. Molecular functions (ATP binding, purine ribonucleoside triphosphate binding, purine nucleoside binding, etc.) accounted for 24.00%. The cellular component (nucleus, intracellular membrane-bounded organelle, membrane-bounded organelle, etc.) was 12.00%. Overall, GO function annotations found that *GRF* mainly functions in molecular pathways.

## Discussion

In our research, the ancestors of land plants contain at least 11 *GRF* genes, which are not much different from the number of existing plants. This indicates that *GRF* genes have not expanded on a large scale. The *GRF* content of ancient species is very small, and it can always cluster in the ancient E branch in the phylogenetic tree. Thus, the *GRF* gene family of land plants originated from the E class. The number of *GRF* in higher plants is significantly elevated than that of ancient species (lower plants), meanwhile, the number of monocots is relatively stable and the number of dicots is more divergent. We found that the ancestors of angiosperms have experienced more gains and the family has expanded. However, the *GRF* gene family of monocots has been shrinking in the course of evolution, while the *GRF* gene family of dicots has not shown a gradual shrinking phenomenon. This points to the different evolutionary processes of monocots and dicots. Combining the results of identification and classification, we found that the low number of *GRF* in lower plants should be caused by excessive loss, rather than a small amount at the beginning.

Our research also showed that *GRF* in foxtail millet is affected by WGD or segmental duplication between 15.07 Mya and 45.97 Mya. The *GRF*s of foxtail millet and other closely related species have strong collinearity and homology. They were mainly subjected to purification selection in the past. These evolutionary phenomena indicate that the evolution of *GRF* is a conservative evolutionary model.

Comparing the qRT-PCR results with transcriptome data showed that 86.67% of the expression trend was consistent. Only 13.33% was inconsistent. This inconsistency has been reported in many literatures (Everaert et al., 2017). Celine Everaert et al. reported that about 85% of the genes showed consistent results between RNA-sequencing and qRT-PCR data. Our result was consistent with that reported. These results indicated that the transcriptome data was reliable.

## Data Availability

The RNA-seq data presented in the study are deposited in the Beijing Institute of Genomics Data Center ( https://bigd.big.ac.cn/) repository, accession number CRA001953.
